# Correction: Kshirsagar et al. Deep Learning Approaches for Prognosis of Automated Skin Disease. *Life* 2022, *12*, 426

**DOI:** 10.3390/life16081216

**Published:** 2026-07-23

**Authors:** Pravin R. Kshirsagar, Hariprasath Manoharan, S. Shitharth, Abdulrhman M. Alshareef, Nabeel Albishry, Praveen Kumar Balachandran

**Affiliations:** 1 Department of Artificial Intelligence, G.H. Raisoni College of Engineering, Nagpur 412207, India; pravinrk88@yahoo.com; 2 Department of Electronics and Communication Engineering, Panimalar Institute of Technology, Poonamallee, Chennai 600123, India; hari13prasath@gmail.com; 3 Department of Computer Science & Engineering, Kebri Dehar University, Kebri Dahar P.O. Box 250, Ethiopia; shitharth.it@gmail.com; 4 Department of Information Systems, Faculty of Computing and Information Technology, King Abdulaziz University, Jeddah 21589, Saudi Arabia; amralshareef@kau.edu.sa; 5 Department of Information Technology, Faculty of Computing and Information Technology, King Abdulaziz University, Jeddah 21589, Saudi Arabia; nalbishry@kau.edu.sa; 6 Department of Electrical and Electronics Engineering, Vardhaman College of Engineering, Hyderabad 501218, India

**Reference Corrections**

In the original publication [1], there were two citation errors in the Introduction Section.

A correction has been made to the Introduction, first paragraph, cited reference “[1] Deepalakshmi, P.; Prudhvi, K.T.; Siri, C.S.; Lavanya, K.; Srinivasu, P.N. Plant Leaf Disease Detection Using CNN Algorithm. *Int. J. Inf. Syst. Model. Des*. **2021**, *12*, 1–21. http://doi.org/10.4018/IJISMD.2021010101.”

This has been replaced with the following reference:

“[1] Alquran, H.; Abu Qasmieh, I.; Alqudah, A.M.; Alhammouri, S.; Alawneh, E.; Abughazaleh, A.; Hasayen, F. The Melanoma Skin Cancer Detection and Classification using Support Vector Machine. In Proceedings of the 2017 IEEE Jordan Conference on Applied Electrical Engineering and Computing Technologies (AEECT), Aqaba, Jordan, 11–13 October 2017; Volume 1, pp. 1–5. https://doi.org/10.1109/AEECT.2017.8257738.”

A correction has been made to the Introduction, third paragraph, cited reference “[4] Kshirsagar, P.; Balakrishnan, N.; Yadav, A.D. Modelling of optimised neural network for classification and prediction of benchmark datasets. *Comput. Methods Biomech. Biomed. Eng. Imaging Vis.* **2020**, *8*, 426–435. https://doi.org/10.1080/21681163.2019.1711457.”

This has been replaced with the following reference:

“[4] Ajith, A.; Goel, V.; Vazirani, P.; Roja, M.M. Digital Dermatology: Skin Disease Detection Model Using Image Processing. In Proceedings of the International Conference on Intelligent Computing and Control Systems (ICICCS), Madurai, India, 15–16 June 2017; Volume 1, pp. 168–173. https://doi.org/10.1109/ICCONS.2017.8250703.”

With this correction, the order of some references has been adjusted accordingly. The authors state that the scientific conclusions are unaffected. This correction was approved by the Academic Editor. The original publication has also been updated.
